# Assessing Vaping Views, Usage, and Vaping-Related Education Among Medical Students: A Pilot Study

**DOI:** 10.7759/cureus.13614

**Published:** 2021-02-28

**Authors:** Tyler Ruppel, Brooke Alexander, Harvey N Mayrovitz

**Affiliations:** 1 Internal Medicine, Nova Southeastern University Dr. Kiran C. Patel College of Osteopathic Medicine, Davie, USA; 2 Medical Education, Nova Southeastern University Dr. Kiran C. Patel College of Allopathic Medicine, Davie, USA

**Keywords:** vaping prevention, e-cigarette and vaping product use associated lung injury (evali), medical education curriculum, electronic cigarette, e-cigarette, vaping, e-smoking

## Abstract

Background

E-cigarette use, or vaping, is known to be associated with potentially life-threatening lung injury, with the Centers for Disease Control and Prevention reporting 2,807 hospitalizations and 68 deaths in the United States due to e-cigarette or vaping-associated lung injury. Vaping is also a risk factor for and is implicated in the spreading of COVID-19. Despite the consequences of vaping, the views and training of medical students regarding vaping is unclear. This study sought to investigate the knowledge and perception of vaping amongst future health care providers.

Methods

An anonymous, online survey was administered to 259 first through fourth year medical students at Nova Southeastern University Dr. Kiran C. Patel College of Osteopathic Medicine via a secure website link in October 2019 and January 2020. The survey consisted of two sections. All participants answered section one, involving nine questions. This first section addressed sociodemographic characteristics, personal views on vaping, rating and impact of vaping medical education, and whether students had ever tried vaping. If students had ever vaped, they proceeded to section two of the survey including nine additional questions. Students that reported no past vaping use ended the survey and did not proceed to section two. Section two focused on evaluating vaping habits of users by questioning age of first usage, use frequency and nature, reasons for first trying and/or continuing to use, plans for quitting, personal impact of use, and current use. Data was analyzed with frequency and percentage distributions.

Results

Most respondents were of age 18-25 years (66.8%) and female (60.2%). Almost all students (96.5%) were aware of the negative health consequences of vaping. More than two thirds of students (68.7%) rated their vaping medical education as inadequate and the majority (76.1%) indicated their medical school curriculum did not impact their view on the matter. Most students (71.0%) reported a more negative stance on vaping due to recent news and media. Of the respondents, over two thirds (37.5%) vaped at least once and were labeled “vapers.” The most commonly stated reason for first trying vaping was recreational (60.8%). Half of vapers (50.5%) admitted to vaping in the past year and most vapers (90.6%) did not think their vaping impacted others. Current vaping use was reported in almost one-third of vapers (32.3%) despite knowing its dangers, and several (6.3%) did not consider vaping dangerous.

Conclusion

The findings from this pilot study conducted at a single medical school indicate possible deficiencies in vaping education, at least as perceived by the respondents and demonstrated by their vaping-related actions. It is unknown if the stated vaping views and practices of these medical students would be positively impacted by better vaping education or if these practices are generalizable to other students. However, the data suggests there is a need to consider more extensive analyses of medical school curriculums with respect to vaping education and training of students in these and related areas. Recommendations to enhance medical school curriculums include vaping-focused respiratory and gastrointestinal lectures, problem-based case studies on vaping, standardized patient encounters, and a community education program taught by medical students.

## Introduction

Vaping refers to inhaling a heated, aerosolized e-liquid from an electronic cigarette device. These devices are portable, discreet, and are associated with a variety of terminologies, such as “e-cigs,” “e-hookahs,” “mods,” “vape pens,” “vapes,” and “tank systems” [1]. The inhaled e-liquid contains nicotine, an addictive neurotoxin, as well as flavored, chemical additives [2]. One such flavoring is diacetyl, a chemical associated with serious lung disease. The aerosol created by e-cigarettes contains benzene, a chemical compound found in car exhaust, as well as heavy metals like nickel, lead, and tin [3]. Surveys of adults in the United States indicate that the highest prevalence of e-cigarette use is among 18- to 29-year-olds [4]. The 2018 National Health Interview Survey reports that 14.9% of adults aged 18 and over admit to using an electronic cigarette at least once, and 3.2% (8.1 million) are current e-cigarette users [5]. Among adult e-cigarette users, reasons for usage most commonly include curiosity, influence of friends or family, and desire to quit or reduce cigarette consumption [6].

Vaping has become prodigiously popular, but not without consequence. The Centers for Disease Control and Prevention (CDC) reports 2,807 hospitalizations and 68 deaths in the United States alone due to vaping product-associated lung injury, largely linked to tetrahydrocannabinol (THC) and vitamin E acetate [7]. According to CDC medical chart data, even non-hospitalized patients experience significant manifestations: 85% experienced respiratory symptoms (e.g., cough, chest pain, and shortness of breath), 57% had gastrointestinal symptoms (e.g., abdominal pain, nausea, vomiting, and diarrhea), and 76% had constitutional symptoms (e.g., fever, chills, and weight loss) [8]. Initial oxygen saturation below 95% while breathing room air was reported among 30% of patients and tachycardia was reported in 40%. Among 34 patients with results reported for initial chest radiographs, 28 (82%) had abnormal findings, and 19 (76%) had bilateral findings. All 28 patients with results reported for chest computed tomography (CT) scans had abnormal findings, including 27 (96%) with bilateral findings.

Although the noxious effects of vaping have come to light, physicians may not be sufficiently able to counsel patients on vaping if there is a lack of education and training on the matter in their respective medical school curriculum. In one study, 92% of providers surveyed were aware of e-cigarettes but frequently cited sources of information about e-cigarettes being patients, news stories, and advertisements, rather than professional, educational resources [9]. These providers had limited knowledge about and comfort with discussing e-cigarettes with patients.

Looking toward the future, medical schools might have an effect in improving this insufficient knowledge amongst physicians by implementing better vaping education into their curriculums. Medical students have an essential role to promote health education amongst the community and their patients, but can only do so with adequate information and supportive research. Moreover, very little is known regarding vaping knowledge and attitudes among medical students. Given that medical students will be the future leaders in medicine, this study intended to examine the education and views of medical students with respect to vaping.

## Materials and methods

The Nova Southeastern University Institutional Review Board approved this study’s protocol and recruitment procedures (IRB #2019-511).

Participants

All first, second, third and fourth-year medical students at Nova Southeastern University Dr. Kiran C. Patel College of Osteopathic Medicine at campuses in Davie and Clearwater, Florida were invited via email to complete an anonymous, online survey that aimed to assess their views, usage, and education regarding vaping. Participation in the study was voluntary and students did not receive direct or indirect benefits from participating in the study.

Survey

The research team collaborated to create all questions in the survey, influenced by various tobacco research survey models. Examples include the Population Assessment of Tobacco and Health (PATH) study created by the US Food and Drug Administration and the National Health Interview Survey created by the National Center for Health Statistics (a sector of the CDC). One objective of the PATH study was to identify and explain between-person differences and within-person changes in tobacco-use patterns [10]. The PATH study also aimed to identify differences and changes in risk perceptions regarding harmful and potentially harmful constituents and identifying other factors that may affect use. Additionally, the National Health Interview Survey implemented questions regarding the knowledge of health risks of smokeless tobacco, attitudes toward and discomfort around smoking, reasons for smoking, age when first smoked, and smoking status one year ago [11]. Paralleling these two models, the research team implemented survey questions that would elicit medical student views on vaping and usage frequency, such as risk perception on the matter, factors influencing use, and reasons for vaping in this specific group of medical students.

The survey consisted of two sections. All participants answered section one (questions one through nine), regardless of e-cigarette usage. This section addressed sociodemographic characteristics, personal views on vaping, and medical education received on vaping. Question nine of section one defined the population of “vapers” as those who answered that they had vaped at least once in their lifetime. These participants proceeded to section two of the survey which included nine additional questions. Those who reported no vaping history did not answer section two of the survey. The set of questions in section two focused on assessing the vaping habits of users, such as age of first usage, number of uses, and characterization of usage, amongst other questions. The specific questions and responses of the survey are listed in the subsequent results section as tables.

The survey was sent via email to all enrolled medical students at Nova Southeastern University Dr. Kiran C. Patel College of Osteopathic Medicine in October 2019. The email contained a link that directed students to a secure Google Forms survey containing the questionnaire. A second email with the survey link was distributed in January 2020. Data collection terminated at the end of January 2020. The Institutional Review Board waived the requirement to obtain written documentation of informed consent. All participants read a participant letter for anonymous surveys which addressed the purpose of the research, possible risks or discomforts, and contact information of the researchers. Participants were explicitly informed of the voluntary nature of their participation and that they were able to exit the survey at any time. If students understood the letter and wanted to participate in the study, they followed the link to the survey. No researchers were present when participants answered the survey.

Data collection and analysis

All data was kept secure on a Google Form Survey Account with a password. Only the principal investigator and co-investigators had access to the account. Responses were downloaded into a spreadsheet without any identifying markers. Data was analyzed using descriptive statistics, including frequency and percentage distributions.

## Results

A total of 1,156 students were invited to participate in the survey, of whom 259 respondents completed the survey (22.4% response rate). All students participated in section one of the survey. Survey respondents were primarily female and within the age group of 18-25 years (Table 1). A majority of students reported that they believed vaping was a danger to one’s health (96.5%). While most students reported the recent/news media impacted their stance on vaping (71.0%), an even higher majority reported their professional school curriculum did not impact their stance on vaping whatsoever (76.1%). Most students rated their professional education on vaping as poor or very poor (68.7%). Students mostly reported that their professional school curriculum did not offer any formal education on vaping (73.4%), but most students reported attempts to educate close family or friends about the negative consequences of vaping (69.9%). Over one-third of medical students admitted to trying vaping in their lifetime (37.5%) (Table 2).

**Table 1:** Demographic Data

	N (Number)	Percentage
1. Age (years)		
18-25	173	66.8%
26-35	84	32.4%
>35	2	0.8%
2. Gender		
Female	156	60.2%
Male	103	39.8%

**Table 2:** Vaping Views

	N (Number)	Percentage
3. Overall, do you think vaping is a danger to a person’s health?		
Yes	250	96.5%
No	9	3.5%
4. Has the recent news/media impacted your stance on vaping?		
Yes- I have a more negative stance on vaping	184	71.0%
Yes- I have a more positive stance on vaping	3	1.2%
No	72	27.8%
5. How would you rate your professional school’s education on the dangers and consequences of vaping?		
Very poor	55	21.2%
Poor	123	47.5%
Fair	54	20.8%
Good	23	8.9%
Excellent	4	1.5%
6. In what way does your professional school curriculum MOST effectively educate students on vaping?		
Lecture format	29	11.2%
Speakers	11	4.2%
Presentations	16	6.2%
Community events	13	5.0%
None; We have had no formal education on vaping	190	73.4%
7. Has your professional school curriculum impacted your stance on vaping?		
Yes- I have a more negative stance on vaping	60	23.2%
Yes- I have a more positive stance on vaping	2	0.8%
No	197	76.1%
8. Have you ever tried to educate close family or friends about the negative consequences of vaping in your lifetime?		
Yes	181	69.9%
No	78	30.1%
9. Have you ever tried vaping in your lifetime?		
Yes	97	37.5%
No	162	62.5%

Students who admitted to past vaping use in section one of the survey were defined as “vapers” and continued to section two of the survey. Thus, only vapers (97 respondents), participated in section two. Most vapers were age 18-25 years at first use of e-cigarettes (79.4%), with the primary reason for first use being recreational/social (60.8%). Vapers mostly stated their frequency of use as “a few times” (60.8%) and half admitted to vaping in the past year (50.5%). Within the past year, 45.4% of vapers characterized their usage as none, 39.2% as light, 8.2% as moderate, and 7.2% as heavy. The primary reason vapers continued to vape was recreational/social (17.5%). Most vapers planned to or wanted to quit vaping within the next year (61.1%). The majority of vapers did not believe their vaping impacted others (90.6%) and almost a third of vapers continued to vape knowing its possible dangers (32.3%) (Table 3).

**Table 3:** Vaping Usage

	N (Number)	Percentage
10. How old were you when you first tried vaping?		
Under 18 years old	9	9.3%
18-25 years old	77	79.4%
26-35 years old	11	11.3%
>35 years old	0	0.0%
11. How many times have you tried vaping in your lifetime?		
A few times	59	60.8%
Sometimes	9	9.3%
Many times	16	16.5%
Frequent user	13	13.4%
12. What is the primary reason you first tried vaping?		
Anxiety or depression	2	2.1%
Peer pressure	6	6.2%
Family member usage	2	2.1%
Recreational/Social	59	60.8%
Pleasurable feeling	5	5.2%
Stress relief/relaxation	8	8.2%
Improved focus and attention	2	2.1%
Appealing flavor/taste	4	4.1%
To quit smoking or using other tobacco products	9	9.3%
13. In the past year, have you vaped, even occasionally?		
Yes	49	50.5%
No	48	49.5%
14. What is the primary reason that you continue to vape?		
Anxiety or depression	2	2.1%
Peer pressure	1	1.0%
Family member usage	0	0.0%
Recreational/Social	17	17.5%
Pleasurable feeling	7	7.2%
Habit	2	2.1%
Stress relief/relaxation	3	3.1%
Improved focus and attention	0	0.0%
Appealing flavor/taste	0	0.0%
To quit smoking or using other tobacco products	2	2.1%
I do not vape currently	63	64.9%
15. How would you characterize your vaping use in the last year?		
None	44	45.4%
Light	38	39.2%
Moderate	8	8.2%
Heavy	7	7.2%
16. Do you plan on quitting or want to quit vaping within the next year or sooner?		
Yes	55	61.1%
No	23	25.6%
Maybe	12	13.3%
17. Do you think your vaping has impacted others around you?		
Yes	9	9.4%
No	87	90.6%
18. Do you continue to vape knowing its possible dangers?		
Yes	31	32.3%
No	59	61.5%
I do not think vaping is dangerous	6	6.3%

## Discussion

Results from this study may suggest the need for formal medical education on vaping and its harmful effects. The study findings also indicate a demand for additional research on the education medical students receive on vaping. Students from the study generally rated their medical education on vaping as inadequate, and most students admitted their professional school curriculum did not impact their stance on the matter. A similar study performed at a medical school in Minnesota reported that 94.8% of medical students believed they did not receive adequate vaping education through their curriculum [12]. The Minnesota report, along with the findings in this study, aligns with research involving practicing providers who did not cite professional, educational resources as their source of information on vaping [9]. Thus, it is imperative that medical students become and stay current on vaping research, knowledge, and guidelines in order to encourage lifestyle modifications and to provide accurate and prompt information to those who vape [13].

A study of 3,030 adult patients reported that only 7.3% discuss e-cigarette usage with their physicians [14]. Since physicians are not discussing the potential harm of e-cigarettes with their patients, it can be inferred that there is a need for more educational opportunities in their medical school years to gain expertise in addressing vaping usage with their patients. Vaping not only induces physiologic bodily harm, but psychological ramifications as well. A survey study regarding adolescent students showed that e-cigarette users had higher levels of depression and suicidality than their non-user counterparts [15]. Thus, the importance of obtaining adequate vaping education and knowledge, as well as discussing its dangers with patients, becomes clear as a means to halting such physiologic and psychologic detriments. Furthermore, since 38.8% of osteopathic physicians and 19.4% of allopathic physicians practice or plan to practice primary care, medical students and their future patients would benefit from more training in preventative medicine and public health issues [16].

Although most medical students were aware of the danger of vaping, 37.5% of students admitted to vaping at least once in their life. This percentage is greater than the 14.9% of adults in the general population that ever vaped in a comparable age group (ages 18 and over) [5]. Additionally, 18.9% of medical students reported vaping in the past year, which is also greater than the 3.2% of current e-cigarette users in the general population [5]. Given the higher rates of vaping among medical students versus the general population, an urgent necessity arises to implement better education about vaping in medical schools.

Moreover, vaping poses a major threat to the COVID-19 pandemic. Comorbidities due to vaping have been shown to render a person more susceptible to COVID-19. For instance, vaping weakens the blood-brain barrier and increases lung epithelial cell permeability, allowing easier viral access to the central nervous system and lungs [17, 18]. Vaping has been implicated not only in the physiological consequences of COVID-19, but also in escalating the spread of the coronavirus pandemic. Sharing of vape devices, which is common amongst users, and transmission of aerosol from these devices, put forth a reasonable assumption that vaping contributes to the spreading of COVID-19 via direct contact or inhalation [19]. Additionally, researchers have found that e-cigarette, or vaping, product associated lung injury (EVALI) on CT imaging closely resembles that of COVID-19 CT lung imaging [19]. It has also been brought into question as to whether previously labeled EVALI CT imaging during the early period of the pandemic may have actually been COVID-19 cases, leading to further spread of the virus [19].

Based on the current findings, it is recommended that medical schools analyze and revise their current curriculum to include vaping and its health effects as educational objectives. It is recognized that medical school curriculums are saturated, and this may require innovation. Such a curriculum may benefit by including respiratory and gastrointestinal lectures on vaping as well as implementing a problem-based learning case study on the matter. Furthermore, it is important for medical students to advocate for vaping-free habits. Students could foster these counseling skills through standardized patient encounters. Additionally, medical schools should create a program targeting community education on vaping products, specifically explaining what is in e-cigarettes and why they are harmful. An example of an educational resource on vaping is the Stanford University Tobacco Prevention Toolkit, an evidence-informed program containing learning modules for tobacco and nicotine education [20]. This program includes discussion prompts, quizzes, factsheets, PowerPoints, and an e-cigarette and vape pen module aimed to prevent the use of tobacco and nicotine among middle and high school students. Surveys completed by 2,889 middle and high school students show that the Toolkit significantly increased student knowledge about e-cigarettes and perceptions that e-cigarettes are harmful and addictive [21]. Medical students could teach this program to middle and high school students as well as the general public through local health fairs. This way, medical students can gain a deeper understanding of electronic cigarette consequences while also having a wholesome and beneficial impact on public health.

There are several limitations in the study that can be addressed in future research. First, the study may not be representative of all medical schools in the United States as only one medical school participated. Second, the 22% response rate also indicates that the results may not be indicative of all medical students from the University. Future research should target numerous medical schools from a wide geographical distribution with varying demographics to ensure a more heterogeneous sample. Third, as occurs with most surveys, the findings were self-reported and, thus, the results could have been skewed due to selection bias. However, studies indicate that self-reported use of tobacco products is a valid representation of actual use [22, 23]. Fourth, some questions were subjective in nature and left up to interpretation by the participant, such as definitions of frequency (a few times, sometimes, many times, frequent user) and vaping usage (none, light, moderate, heavy), which could have resulted in varied interpretation between students. Lastly, discrepant responding limits this research. A slight discrepancy exists between past-year vapers who report they no longer vape. For example, question 14 indicated that 64.9% did not currently vape; however, 45.5% reported no use in question 15. This reflects a difference of 21 out of 97 students and is a result of discrepant responding of participants. Similarly, a difference arises between percentages of past-year use and whether the participant had quit vaping. Forty-four of 97 students reported no past-year use in question 15, leaving 53 students who indicated some level of past-year use. However, when reporting on quitting in question 16, 55 of 97 students planned to or wanted to quit in the next year. This demonstrates a difference of two students and again is a result of discrepant responding.

## Conclusions

According to the results of this study, although most medical students view vaping as a health hazard, students receive little to no formal medical education about the detriments of vaping. Students cite that even the news and media have more of an impact on their views than their own medical education. Furthermore, the vaping usage of medical students surpasses the general population with over one-third of medical students trying e-cigarettes at least once as well as up to 18% of students being past-year users. This study suggests a clear necessity for future research analyzing the vaping education in medical school curriculums. Curriculums deemed inadequate could benefit from implementing electronic cigarette education to promote healthy living amongst medical students, their future patients, and the entire community.
